# The ‘diamond concept’ for long bone non-union management

**DOI:** 10.1186/s10195-019-0528-0

**Published:** 2019-04-11

**Authors:** Paul Andrzejowski, Peter V. Giannoudis

**Affiliations:** Academic Department of Trauma & Orthopaedics, School of Medicine, University of Leeds, Clarendon Wing, Floor D, Great George Street, Leeds General Infirmary, Leeds, LS1 3EX UK

**Keywords:** Diamond concept, Bone healing, Long bone, Non-union, Mesenchymal stem cells

## Abstract

Long bone non-union continues to be a significant worldwide problem. Since its inception over a decade ago, the ‘diamond concept’, a conceptual framework of what is essential for a successful bone healing response, has gained great acceptance for assessing and planning the management of fracture non-unions. Herein, we discuss the epidemiology of non-unions, the basic science of bone healing in the context of the diamond concept, the currently available results and areas for future research.

## Introduction

Since its inception, the diamond concept has proven itself to be an important framework for understanding the minimal requirements for fracture healing. Moreover, it has shown itself to be particularly useful when planning surgical management of fracture non-union of both upper and lower extremities [1–13].

Non-union has been defined in various ways, with a 55% disagreement amongst clinicians on timing [14]. The US Federal Drug Administration council defines it as ‘failure to achieve union by 9 months since the injury, and for which there has been no signs of healing for 3 months’. Others, however, have recommended that for long bones this should be revised to a period of 6 months if no evidence of radiological fracture healing is present [15]. Instability at the fracture site in true non-union is often associated with ongoing pain, and as such, clinical signs are as important in diagnosis as the radiological examination [14, 16].

Well-vascularised fracture sites with abundant fracture haematoma but an unstable mechanical environment will usually develop ‘hypertrophic’ non-union, whereas impaired blood supply in combination with local strain concentration has been suggested to lead to ‘atrophic’ non-union [17]. These definitions are based on the radiographic appearance of non-union [17]. The presence or absence of infection is also important in terms of classification, which can further complicate the clinical picture and treatment modality [18].

Incidence of non-union has been variably reported in the literature, depending on study size, patient demographics, injury location and severity and method of treatment, from anywhere between 2 and 30% [19] with an estimated 100,000 episodes of fracture non-union per year in the USA [16]. A recent study from Australia on 853 patients showed overall 8% of patients who had fractures, being admitted to hospital per year for fracture healing complications [20]. However, a recent much larger population-based study done in Scotland showed lower overall incidence than previously reported, at 1.9% in the adult population, with incidence of non-union for pelvis and femur fractures of 13 per 1000, humerus of 30 per 1000 and tibia of 55 per 1000; incidence was seen to peak in the 25–44-year age group [21]. This comes with significant financial implications, with reported overall costs between £21,183 and £33,752 per patient [22, 23].

### Risk of non-union

Risks of non-union can be defined as patient dependent and independent, as well as local and systemic, some of which can be modified to enhance fracture healing.

A recent systematic review into risk stratification showed that quality of evidence quantifying relative risks is variable, ranging from level 1 to level 5 evidence. Table 1 illustrates a summary of existing risk factors [16, 24–26].

**Table 1:** Risk factors for non-union

Patient dependent		Patient independent
Modifiable	Non-modifiable	
Smoking	Age	Open reduction (poor quality of primary ORIF)^a^
Alcohol	Male gender	Open fracture (more bone loss and soft tissue injury)
Nutritional deficiency (including vitamin D)	Genetic predisposition^b^	Wedge and multi-fragmentary fracture pattern
High BMI	Diabetes (metabolic disease)	Initial displacement
	Peripheral vascular disease	Compartment syndrome^a^
	Osteoporosis	Affected bone: highest in tibia
	Chronic inflammatory disease	Fracture site in relation to vascularisation zone
	Renal insufficiency	Presence of fracture gap post-surgery^a^
	Insulin^a^	Poor mechanical stability by initial implant^a^
	Opiates^a^	Infection^a^
	NSAIDs^a^	
	Steroids^a^	
	Antibiotics^a^	
	Anticoagulants^a^	
	Chemotherapeutics^a^	

^a^Potentially modifiable, ^b^Inconclusive—under research

Attempts have been made to develop scoring systems to predict the risk of early non-union, including the Non-Union Scoring System (NUSS) [27] and the Moghaddam risk score [28]. Some authors have suggested that the appropriate treatment modality of non-union should be based on the severity of the non-union scoring system used.

### Fracture healing and the diamond concept

A successful fracture healing response is dependent on the biological environment at the fracture site (availability of molecular mediators, progenitor cells and matrix, immunoregulatory cells amongst others) and an optimum mechanical environment that provides the fracture site with adequate stability, facilitating the evolution of a physiological process leading to a successful bone repair response. In general terms, there are two mechanisms by which bone can heal, which are influenced by the local mechanical fracture environment. Direct, or primary cortical bone healing occurs where there is ‘absolute stability’ of bony surfaces with close contact of less than 0.15 mm, and minimal inter-fragmentary strains of less than 2%; this can only be achieved by compression lag screw or compression plating [16, 29–31]. The second mechanism is by indirect, or secondary bone healing which is facilitated by relative stability. Overall, the phases of fracture repair are broadly divided into fracture haematoma formation, inflammation, cellular proliferation and differentiation, and remodelling [32, 33].

**Fig. 1:**
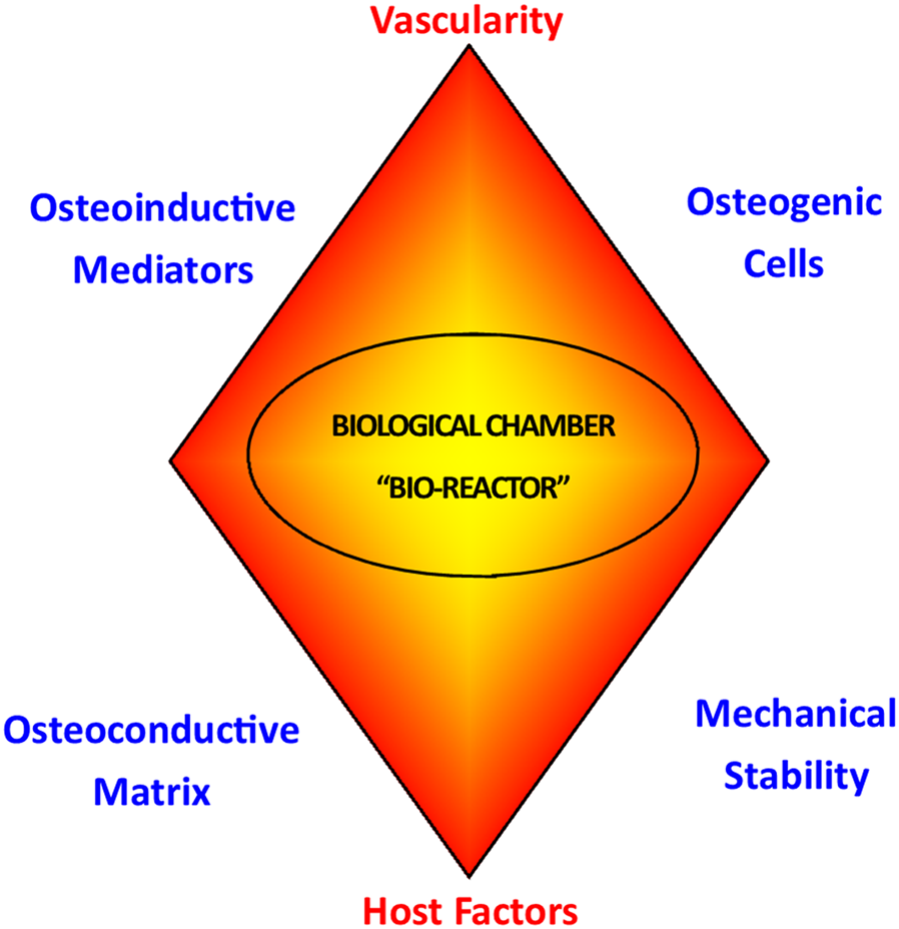
Illustration of the ‘diamond concept’ of bone healing

The ‘diamond concept’, being a conceptual framework for a successful bone repair response, gives equal importance to mechanical stability and the biological environment. Moreover, adequate bone vascularity and the physiological state of the host are thought to be essential within this framework of fracture repair. A deficit in the biological environment or the mechanical environment, or failure to appreciate the comorbidities of the host and the lack of vascularity can all lead to an impaired fracture healing response (non-union). Overall, the diamond concept refers to the availability of osteoinductive mediators, osteogenic cells, an osteoconductive matrix (scaffold), optimum mechanical environment, adequate vascularity, and addressing any existing comorbidities of the host (Fig. 1) [1, 2]. The important constituents of the diamond concept are discussed in more detail below.

#### Osteoinductive mediators

**Fig. 2:**
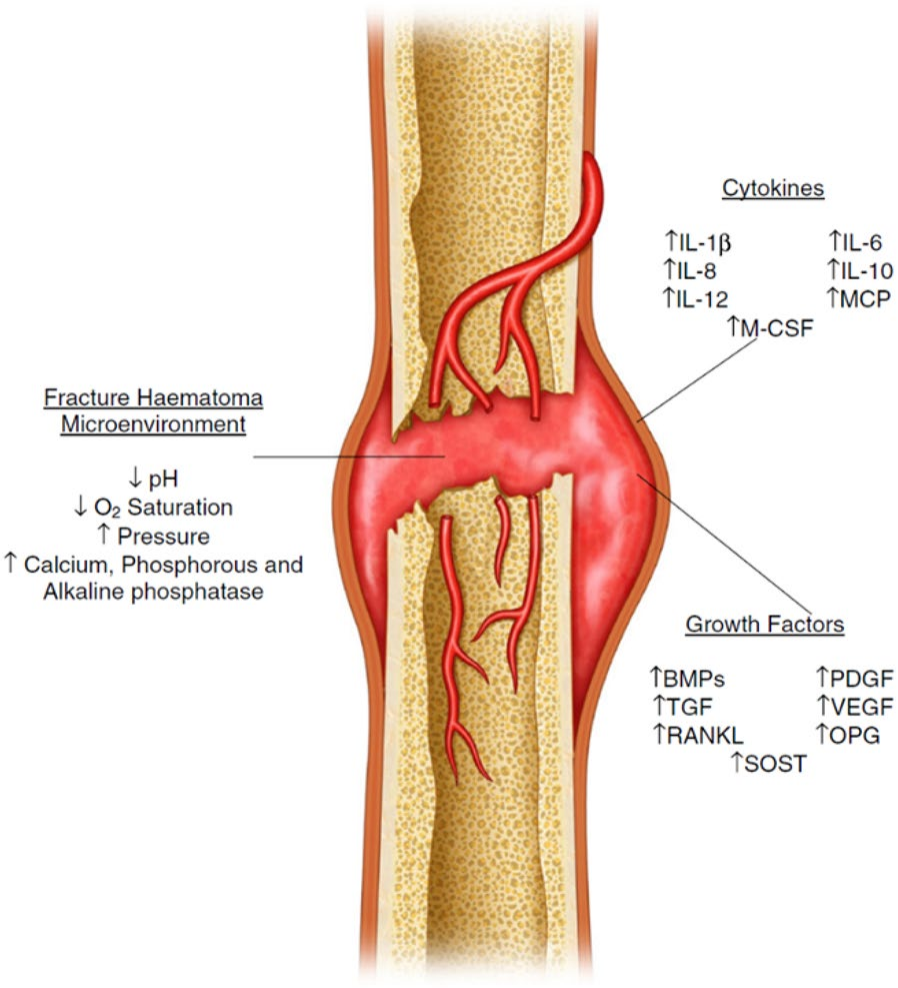
Diagrammatic representation of fracture haematoma composition. Key: *IL* interleukin, *MCP* monocyte chemoattractive protein, *M-CSF* monocyte colony-stimulating factor, *BMP* bone morphogenic protein, *PDGF* platelet-derived growth factor, *VEGF* vascular endothelial growth factor, *RANKL* receptor activator of nuclear factor kappa-B ligand, *OPG* osteoprotegerin, *SOST* sclerostin (Adapted from Walters et al. [34] used with permission)

Initial bleeding following fracture initiates the coagulation cascade; this leads to development of a fracture haematoma [34]. This contains platelets and macrophages, which release a series of cytokines (cell signalling molecules) of different types, stimulating a cascade of events to initiate healing. These include proinflammatory interleukins 1, 6, 8, 10 and 12, tumour necrosis factor-a (TNFa), activated protein C (APC), monocyte chemoattractive protein (MCP), macrophage colony-stimulating factor (M-CSF), receptor activator of nuclear factor kappa B ligand (RANKL) and osteoprogenin (OPG) [1, 34, 35]. Metalloproteinases and angiogenic factors such as vascular endothelial growth factor (VEGF) also play an important role in the overall bone repair process [1, 34, 35]. However, the most important mediators released having a direct effect on progenitor cells to undergo the process of mitogenesis and osteoblastic differentiation include platelet-derived growth factor (PDGF), fibroblast growth factor (FGF), insulin-like growth factor (IGF) and transforming growth factor beta (TGFβ) proteins, which include bone morphogenic protein (BMP)-2, 4, 6 and 7 (Fig. 2) [1, 34, 35].

#### Osteogenic cells

**Fig. 3:**
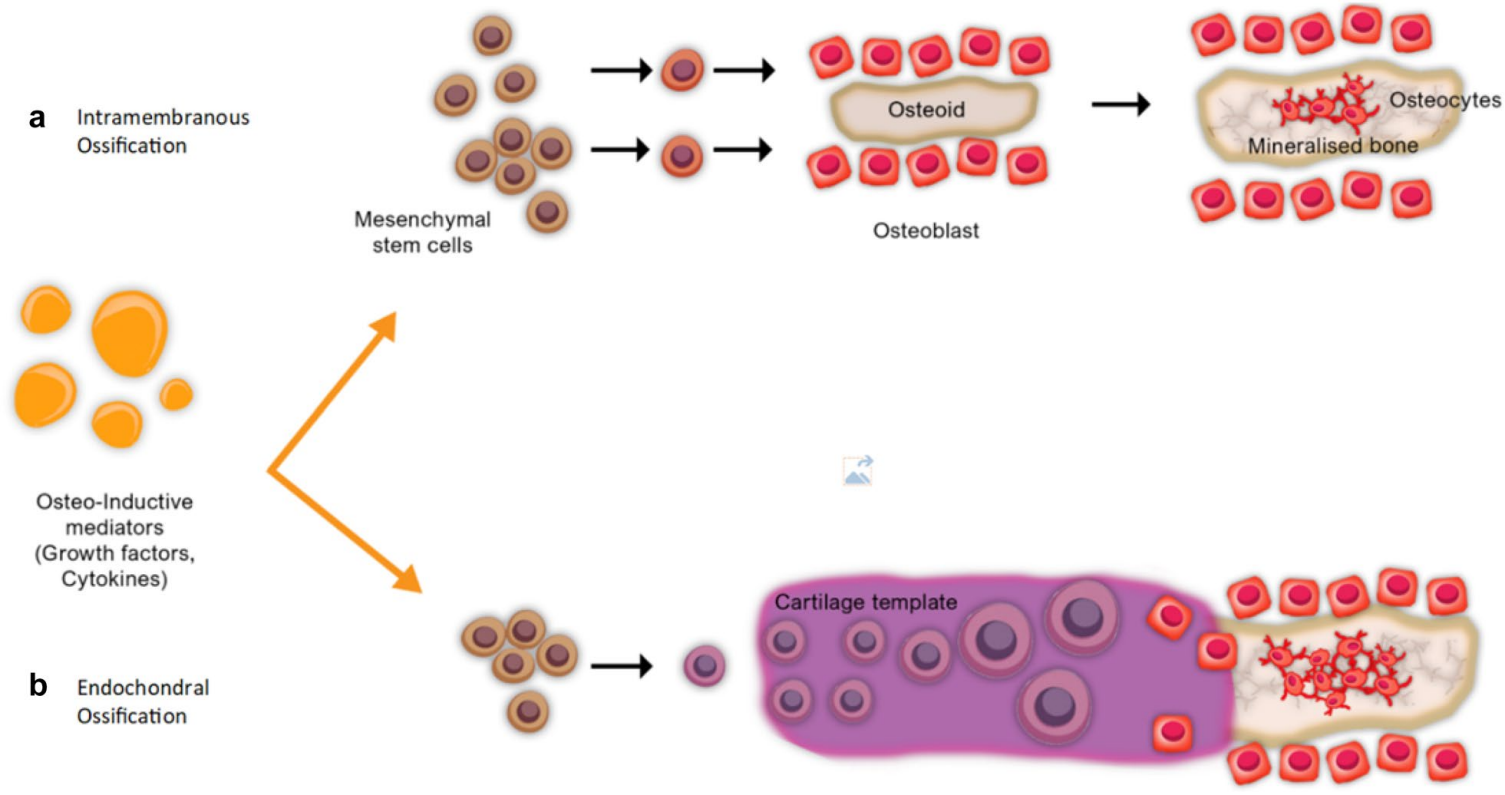
Diagrammatic representation of ossification: **a** Intramembranous ossification. Osteoinductive mediators induce osteogenic MSCs to differentiate into osteoblasts, which lay down osteoid (collagen-1 rich); this mineralises to form an ossification centre, whence mineralisation extends. There is terminal differentiation into osteocytes, becoming entombed in the bone matrix. **b** Endochondral ossification. Osteoinductive mediators induce osteogenic MSCs to differentiate into chondrocytes; a cartilage matrix is secreted which forms the template for endochondral bone formation. Chondrocytes then undergo hypertrophic differentiation and mineralise the surrounding matrix. They eventually undergo apoptosis—resulting in vascular invasion. Invading blood vessels convey osteoblasts which form bone on the cartilage template [46]

Osteogenic cells, which comprise both committed osteoprogenitor cells from the periosteum as well as undifferentiated multipotent stem cells (MSCs) from bone marrow and endothelial progenitor cells, are also activated according to the local fracture environment in the haematoma [1]. The cytokine release leads to an ensuing inflammatory phase, characterised by increased blood flow and vascular permeability, and chemotaxis with activation of the complement cascade. Osteoclasts and fibroblasts initiate conversion of haematoma into granulation tissue, laying down a fibrin meshwork which is then invaded by a new capillary network allowing further MSC migration. Following activation, cytokines are also released by endothelial cells, MSCs, chondrocytes, osteocytes and osteoblasts themselves [1, 15, 34, 36]. This is followed by the proliferation and differentiation of MSCs, leading to simultaneous hard and soft callus formation, which is highly influenced by the mechanical micro-environment and fracture biology [29, 32, 37]. Higher oxygen tension at periosteal surfaces distal to the fracture site, as well as other factors, encourages preferential MSC differentiation into osteoblasts [16]. In the peripheral (cortical) zone, osteocalcin initiates periosteal osteoblasts to produce type 1 collagen, leading to intramembranous ossification (hard callus). In central (medullary) zones, MSCs develop into chondrocytes, initially laying down type 2 collagen (soft callus) known as endochondral ossification; by week 3, increasing osteocalcin induces calcification and hard callus formation here too (Fig. 3). Mineralisation of fracture callus into an osteoid-type matrix and type 1 collagen fibrils leads to bridging of the fracture site and disordered ‘woven bone’ formation [31, 38–41]. Critical in guiding this process are the BMPs, which are responsible for inducing osteogenic activity in mesenchymal stem cells and maturation of lamellar bone, as well as helping coordinate osteoclastic activity [32, 42–44]. Inhibitory and fibrinolytic molecules also play a key role in regulating the process, without which bone healing has shown to be delayed [32, 35, 45]. This is followed by a period of remodelling by bone multicellular units (BMUs) in a process of activation, resorption, reversal and formation, taking at least 6 months to complete. The disordered woven bone, which is comparatively weak, develops into stronger, organised lamellar bone following in general the principles of Wolff’s law, who showed that the trabecular pattern of bone corresponds to the mechanical stresses placed upon it [46–48].

#### Extracellular osteoconductive matrix (scaffold)

An osteoconductive extracellular matrix, acting as a scaffold and promoting migration and adhesion of osteoinductive and osteogenic cells to the fracture site, is essential for fracture healing. Where there is good apposition of bone, necrotic bone at the fracture site serves this purpose. If there is insufficient ‘natural’ scaffold, then autograft, or allograft demineralised bone matrix (DBM), which also has inherent osteoinductive capability thanks to retained growth factors including BMP, can be used when treating non-union of bone defects [1, 32, 42, 43, 49, 50].

#### Mechanical environment

Evidence suggests that cells are able to sense the surrounding mechanical environment, through electrochemical signals generated by fluid shift within canaliculae (osteocytes), and also other cell types have cell membrane mechanoreceptors and direct connections between the cell nucleus and local cytoskeleton, which are further influenced by the chemical environment and cellular signalling molecules [37]. Based on studies of cells in culture, cellular development has been shown to be greatly influenced by local mechanics, with the mechanical and physiological environment impacting significantly upon subsequent lineage differentiation of multi-potent mesenchymal stem cells. In the presence of appropriate growth factors, tension encourages fibroblasts, shear encourages chondroblasts, and a combination of compression/distraction encourages osteoblasts—reflecting the mechanical environment in which the cells usually develop [29].

Strain, defined as extension per unit length in relation to the force applied, reflecting loading and micromotion at the fracture site, is important to initiate healing, as discussed by Perren [17, 30]. Axial micromotion seems to stimulate fracture healing in the early stages; by 8 weeks, this relationship reverses, being reflected in normal healing by increasing callus stiffness, which naturally aims to decrease movement at this stage [29, 30]. Low strain rates promote intramembranous ossification, but endochondral ossification is more likely to be initiated if the strain rate is increased. When strain is increased too far, however, increased differentiation down the soft tissue lineage pathway predominates, leading to delayed or non-union [31]. For ossification to occur, the fracture gap must have reduced to an appropriate level, impacted in turn by the relative stiffness of the tissues around it. Experiments have shown that this should be ideally less than 2 mm and certainly less than 6 mm, above which little callus is seen to form [31, 38].

#### Vascularity and host factors

If vascular supply or fracture haematoma is compromised or lost, there is a higher risk of non-union, as insufficient osteoinductive and osteogenic cells will be available at the fracture site to initiate osteogenesis, remodelling and healing [2, 45]. The chance of this significantly increases in high-energy and open fractures, or in primary surgical repair where the fracture biology, periosteum and soft tissue envelope are not respected [24, 51]. Periosteum, as well as providing critical blood supply, also has unique regenerative potential [52, 53]. Similarly, if there is altered systemic host (patient) physiology or comorbidities, this will also impact healing potential [2, 24, 45, 51, 52].

### Biological chamber

The concept of the biological chamber is based on the need for containment; For instance, where a long bone non-union case has been managed with the diamond principle, one has to appreciate that any biological enhancement that was placed at the site of non-union must be contained locally so that the maximum effect will be exerted. It is the development of a chamber which allows an influx of biological activities to promote a healing response in a timely fashion. In a sense, what we are referring to is the development of a ‘bioreactor’. Confinement of the treatment selected for non-union can be achieved with modification of soft tissues, biological membranes, sealants etc. This is especially important when one considers the relative mean retention times of these ingredients [2, 54].

## Methods

Search criteria: Ovid SP was used to search Embase Classic and Embase databases, as well as Ovid Medline. Search term under ‘all headings’: ‘diamond concept’. Papers which were not original and not discussing long bones, not using human subjects, not in the English language and review articles and letters were excluded from the study; one poor-quality case report was excluded from the study also. However, review articles were downloaded and used for reference purposes.

Each paper was systematically searched for: area of treatment, level of evidence, study size (*n*), objectives, study type, patient characteristics, methods, non-union risk profile, assessment of union, length of follow-up, time to union, and outcomes: radiological and clinical union, complications, microbiology results, and any risk factors which correlated with non-union, and documented in tabulated form, allowing straightforward reference for discussion.

## Results

Overall, we found ten studies which met our inclusion criteria, including five retrospective cohort or case–control studies [4, 6, 9, 12, 13], three prospective cohort studies [10, 11, 55] and two case reports [7, 8]. One study was excluded (case report by Dilogo et al. 2017), as it was unclear whether this patient had achieved confirmed union at the time of reporting. The total number of patients included in these studies was 548. Overall success in treating non-union, when rigorously applying all aspects of the diamond concept, was 89–100%. When fewer of the principles and augmented elements of the ‘diamond’ were applied and depending on fracture type and location, overall success ranged from 44 to 90% (Table 2).

**Table 2:** Summary of literature review

Study, year	Area of treatment	Level of evidence	Study size (*n*)	Objectives, study type, patient characteristics, methods, assessment of union	Follow-up (months)	Healing (months)	Outcomes
Calori, 2013 [9]	Forearm: 19 radius 26 ulna 6 both 1 Monteggia	3	52	To assess efficacy of ‘monotherapy’ versus ‘polytherapy’ (diamond concept) in non-union Retrospective cohort study: Mono: *n* = 33, poly: *n* = 19 Non-union risk profiles: NUSS score: mono: 36 ± 8.88, poly: 58.84 ± 9.44 Mx: Both: debridement ± metalwork revision (stability) Monotherapy Mx: ABG/MSC/BMP7/ABG or synthetic bone matrix Polytherapy Mx: BMP7 + MSCs + ABG (53%) or synthetic bone matrix	12	Clinical: 3.65 Radiological: 6.18	Radiological union: mono: 63.6%, poly ‘diamond’: 89.5% Time to union: Clinical union (months):* mono: 5.29, poly ‘diamond’: 3.65 Radiological union (months):* mono 8.43, poly ‘diamond’: 6.18
Miska, 2016 [12]	Humerus	3	50	To assess if individualising treatment for non-union based on diamond concept and risk score is effective Retrospective cohort study: n = 50, Age 51.3 years (14–88) Non-union risk profiles: Mean Moghaddam scores: treated with BMP-7: 16.5, without BMP-7: 12.6, *p* = 0.83 Previous interventions: mean 1.5 (1–8) Mx: According to risk, individual aspects of diamond addressed: Low–moderate risk: debridement and re-osteosynthesis only Infected cases: two-stage Masquelet If large defects > 2 cm or devitalised tissue: Debridement ± BMP-7 ± RIA (MSCs, scaffold) from femur or iliac crest ABG ± re-osteosynthesis with stable plate (90%)	≥ 12	6	Radiological union: overall: 80.4%, those who received BMP-7: 6/8 (75%) Patients successful in union much younger (46.6 ± 17.5 versus 62.4 ± 16.5 years), *p* = 0.031* Risk scores: did not predict non-union Only 6 patients managed with plate/cancellous bone/BMP-7. No MSCs used. No specific data given for these in terms of union rates compared with others
Giannoudis, 2015 [3]	Multiple site: femur (54.68%) tibia (34.38%) radius (3.13%) clavicle (3.13%)	3	64	To assess efficacy of long bone non-union treated with the ‘diamond concept’ Prospective cohort study: *n* = 64. Age 45 (17–83) Non-union risk profiles: All had at least 1 significant comorbidity, 65.63% suffered high-energy initial injury 17% initially open fractures. ≥ 1 previous interventions (1–5) in 43.75% Mx: Debridement + metalwork revision + Iliac crest BMAC (MSCs) + BMP-2 (5%) or BMP-7 (95%) + RIA contralateral femur for ABG (scaffold and osteogenic cells)	12 (12–32)	6 (3–12)	Radiological union: 63/64 (98%) by 12 months
Giannoudis, 2013 [4]	Femur: subtrochanteric	3	14	To assess clinical outcome of diamond concept in patients with IM nails in non-union surgery Retrospective cohort study: *n* = 14. Age 65 (33–92) High-energy fracture in 4 patients Mx: Debridement + blade plate or revision IM nail + RIA contralateral femur for ABG (scaffold and osteogenic cells) + BMP-7 + BMAC (MSCs) from iliac crest, watertight closure in layers	26 (16–48)	6.8 (5–12)	Using the ‘complete diamond’ for all patients: Radiological union: 13/14 (92%)
Goff, 2014 [8]	Proximal femur (intertrochanteric)	4	1	To assess efficacy of diamond concept in challenging case of infected femoral non-union Case report *n* = 1, Age 31, male. Hx and risk profile: 11 months following well-sited DHS for high-energy intertrochanteric fracture (RTA). Fit and healthy, non-smoker Mx: Masquelet technique: Stage 2 (at 2 months): Debridement, RIA contralateral femur for ABG (scaffold and osteogenic cells) + BMP-7 composite graft + removal of ex-fix and application of blade plate	42	6	Radiological union: 6 months Clinical union: 6 months
Haubruck, 2018 [6]	Lower limb 69 femur 87 tibia	3	156	To assess which is superior in non-union surgery: BMP-2 or BMP-7 Retrospective case–control study: n = 156, Age 51 (18–64) Mx: One stage: limited + no infection Two stage: (Masquelet technique): significant bone loss ± signs of infection Both: debridement + metalwork revision + ABG (MSCs, scaffold) + BMP-2 or BMP-7 No concentrated BMA used	≥12	No data provided	Radiological union:* BMP-2: 42/46 (91%), BMP-7: 64/110 (58%) Femur union: BMP-2: 11/14 (79%), BMP-7: 33/55 (60%) Tibia union:* BMP-2: 31/32 (97%), BMP-7:24/55 (44%) One stage: BMP-2: 7/8 (88%), BMP-7: 35/50 (70%) Two stage:* BMP-2: 35/38 (92%), BMP-7: 29/60 (48%)
Ollivier, 2015 [13]	Tibia	3	20	To assess whether bone grafting essential as part of diamond concept for recalcitrant tibia non-union Retrospective cohort study: n = 20, Age 46.8 (21–78) Non-union risk profiles: open fractures: 8, smokers: 5. Mx: Debridement + metalwork revision + implant composite graft (BMP and injectable rCPBS) No MSCs/BMAC used	14 ± 2.7 (3–9)	5 ± 2.3 (3–9)	Radiological union: 18/20 (90%): 12 had consolidated by 3 months, and 18 by 6 months Micro: nil significant Did not use all parts of diamond concept: no osteogenic MSC cells harvested or implanted
Moghaddam, 2015 [10]	Tibia	3	102	To assess outcomes of single-stage (G1) versus two-stage (Masquelet) repair (G2) methods in tibial non-union Prospective cohort study: *n*: G1 = 52, G2 = 50. Age 47 ± 13.1 (15–76) Previous interventions: G1: 3.2, G2: 6.7 Non-union risk profiles: open fractures: 44, fracture gap: 2.6 ± 3.4 cm Moghaddam score:* G1: 13.8 ± 8.5, G2: 19.5 ± 9.5 NUSS score:* G1: 38.3 ± 11.7, G2: 48.2 ± 10.3 Mx: G1: bone loss < 1 cm (mean 0.9 cm) + no infection G2: bone loss > 1 cm (mean 4.0 cm) + signs of infection (Masquelet technique) Both: debridement ± metalwork revision (97%), RIA (MSCs, scaffold) from femur (77%) or iliac crest ABG (23%) + BMP-7 + tricalcium phosphate	12	G1: 6.9 ± 3.1 G2:* 8.6 ± 2.9 Overall: 7.8 ± 3.1	Radiological union: G1: 84%, G2: 80%
Douras, 2018 [7]	Ankle: medial malleolus	4	1	To assess efficacy of diamond concept in medial malleolus non-union Case report, *n* = 1 Age 20 Hx: 8 months following: Gustilo IIIb open bimalleolar fracture dislocation ORIF and free flap Non-union mx: Debridement + metalwork revision with cancellous screws and locking plate + BMAC (MSCs) and ABG (scaffold, MSCs) from iliac crest + BMP-2	12	6	Radiological union: by 6 months Clinical union: by 3–6 months (fully WB) Complications: none

*ABG* autologous bone graft, *BMP* bone morphogenic protein, *RIA* reamer/irrigator/aspirator, *MSCs* mesenchymal stem cells, *BMAC* bone marrow aspirate concentrate, *rCPBS* resorbable calcium phosphate bone substitute

Key: *n* patient number, *Mx* Management, *p < 0.05

## Discussion

The search for the best approach to treat long bone non-union is ongoing. Particularly recalcitrant ones are more difficult to manage, with long-lasting treatment and high cost implications. The diamond concept approach offers a new paradigm for their management. In addition to correcting the mechanical environment, a potent biological stimulus is provided locally by addition of a scaffold, growth factors and multipotent stem cells whilst respecting the local blood supply and fracture biology. Moreover, patient-related comorbidities must be addressed to overcome inherent limitations of the host physiological processes.

In regard to upper limb studies, Calori et al. [9] performed a study on 54 patients with upper limb non-union, comparing polytherapy ‘diamond concept’ versus monotherapy for non-unions. Patients treated with polytherapy [BMP-7, MSCs, synthetic or autologous bone graft (ABG), re-osteosynthesis] had worse cases of non-union to begin with. Statistical analysis demonstrated superiority of polytherapy over monotherapy in clinical, radiological and functional outcomes, and a higher percentage (89 versus 63%) went onto union. Despite limitations of fracture diversity, sample size and retrospective nature of the study, these results are compelling. Miska et al. [12] looked at 50 patients with humeral non-union, managed depending on their risk profile with various aspects of the diamond concept, with only 6 patients having the full spectrum of the diamond addressed. These were treated using angular stable plating (mechanical stability), autologous bone graft (MSCs and scaffold) and BMP-7 application (osteoinductive agent). Overall union rates of 80% were seen, however it is not clear how outcomes in the ‘diamond concept’ group faired in comparison with the others.

Haubruck et al. [6] treated 156 patients with lower limb non-unions (69 femurs and 87 tibias), comparing BMP-2 with BMP-7 for single- and two-stage revisions, using ABG to provide a scaffold and MSCs, and performed re-osteosynthesis to enhance stability. Overall union rates were 91% with BMP-2 and 58% for BMP-7 (*p* < 0.001), with similar rates of overall healing between femur and tibia when BMP groups were combined, at 64 and 63%, respectively, but in the BMP-2 group these were 79% and 97%, respectively, illustrating a good potential of the diamond concept.

Giannoudis et al. [4] published a retrospective cohort study of 14 patients with subtrochanteric femoral non-unions, including four open fractures. Excellent outcome was achieved using all four principles in the diamond concept: debridement, blade plate or revision IM nail (mechanical stability), RIA (reamer/irrigator/aspirator) from the contralateral femur for ABG to use primarily as a scaffold, BMP-7 and bone marrow aspirate concentrate (BMAC) to provide MSCs from the iliac crest, with watertight closure in layers to ensure containment of bioactive material in the ‘biological chamber’. Overall union rate was 90%. This study also highlighted the presence of varus mal-alignment of fixed acute fractures, emphasising that failure of mechanical stability was successfully corrected as part of ‘diamond concept’-focused management. Goff [8] also reported an excellent outcome when applying all aspects of the diamond concept in treating delayed femoral intertrochanteric non-union, by using a two-stage modified Masquelet technique augmented with BMP-7 and RIA used to supply ABG. Giannoudis et al. [55] performed a further study, looking at fracture non-union from all sites (upper and lower limb). Applying the diamond concept to treat 64 patients of whom 65% suffered high-energy injuries, they obtained a union rate of 98% by 12 months, again showing the potency of the diamond concept.

Moghaddam et al. [10] in a study of 88 patients with subtrochanteric femoral non-unions, including 21% with open fractures, applied all elements of the diamond concept in patients deemed to be in a high-risk group for healing as part of a single- or two-stage procedure: RIA samples from the femur or ABG from the iliac crest used as a source of MSCs as well as a scaffold material, and some also supplemented with BMP-7 and tricalcium phosphate. Seventy-two patients received full ‘diamond concept’ augmentation, 13 cases only had ABG, and 3 only had revision of metalwork. No complete ‘diamond concept’ group-specific outcome data are provided. Overall, 69 patients (78%) achieved good healing when applying this methodology, with union in single-stage procedures higher (95.1%) compared with two-stage (63.8%) overall. Rates of union were significantly higher in the femoral diaphysis compared with distal femur (84 versus 70%, respectively), especially when managed using an intramedullary nail. Larger defects (5–10 cm) in the diaphysis managed with a two-stage procedure had poor healing rates (58%), with smoking having a significant impact. Only in cases where the defect did not cross the entire diameter and an osseous bridge was still present did healing occur. This study demonstrates that poor vascularity and difficulty in achieving mechanical stability lead to impaired fracture healing, illustrating their importance for inclusion in the diamond.

Moghaddam et al. [11] also investigated the treatment of tibial non-unions, with successful union of 84% in single-stage (group 1) and 80% in two-stage (group 2) procedures, augmenting elements of the diamond concept according to risk profile, with 76.8% of patients overall receiving a combination of RIA, BMP-7 and tricalcium phosphate, in addition to re-osteosynthesis. Again, no ‘complete diamond’ group-specific data are provided. Results show that applying ideas of the diamond concept based on risk profile when treating both fracture types led to acceptable union rates. Despite having worse initial starting position, patients in G2 had similar outcome afterwards. Authors suggest that a higher number of previous surgeries in G2 patients (mean 3.4 versus 2.4) contributed to worse healing due to scar tissue impeding blood flow, however provide no absolute values or multivariate analysis with *p* values to substantiate the outcomes of this subgroup. G1 and G2 groups both fell into the ‘medium’ risk category for non-union on the Moghaddam prediction score, as well as the NUSS, and behaved as would have been expected; also patients in G2 who had higher overall scores took longer to heal. Microbiology results suggest that, in previously undetected atrophic non-unions, low-grade infection may be the cause, and that in these cases, the Masquelet technique can be used effectively. Results also suggest that antibiotic osteitis prophylaxis was beneficial in the G1 group treated with gentamicin-coated nails.

Douras [7] presented a case study in which the full diamond constituents were applied for a medial malleolus non-union, and achieved full union by 6 months, with the patient back to normal function, showing that the technique is also effective in ankle fracture non-union.

Olivier [13] performed a study on 20 patients with recalcitrant tibial non-union, which aimed to apply the diamond concept principles in a different way. As well as debridement and re-osteosynthesis, a composite graft using synthetic resorbable calcium phosphate bone substitute combined with BMP was used instead of ABG, which may be useful in patients with poor bone stock. It is worth noting, however, that no augmentation using stem cells was used. Union rates were 90%, suggesting that it could be a safe and effective alternative where ABG is not possible.

The studies discussed here represent all of the literature which specifically refers to using the ‘diamond concept’ as a principle in guiding management decisions in long bone non-union. As one can see, where all of the diamond concept principles are clearly adhered to and elements of this augmented during surgery, there are excellent rates of union after treatment for upper and lower limbs non-unions. Biological and mechanical environment, systemic factors and local blood supply as well as fracture pattern, location and frequency of previous intervention and stability of implants used all could predict outcome. However, only a few of the above studies use bone marrow aspirate concentrate (BMAC) as a direct means of concentrating mesenchymal stem cells to act as osteoprogenitors, with most relying on the MSCs present in RIA or autologous bone grafting from the iliac crest. In our experience, we have found that the RIA process washes most of the MSCs out of the bone stock, reducing its utility as a source of potent osteogenic cells. As we have previously published, after the age of 55, even the iliac crest becomes less useful as a source of MSCs with a more ‘yellow’ appearance and fewer stem cells to aid union [56]. We believe that, in compromised cases, the use of BMAC to supply a reliable source of MSCs as part of the diamond can help to achieve good results.

The evidence available to show that using ‘polytherapy’ with the diamond concept is preferable over ‘monotherapy’ is convincing, but numbers are still small, as also discussed by Calori et al. [57]. Some of the studies presented have only augmented all aspects of the diamond concept for high-risk patients, which may be obscuring the picture. Further studies in the future, of prospective randomised nature, would throw more light on this matter.
